# Hospital-Based Models of Immunization for High-Risk Subjects in Lombardy (Italy): A Region-Wide Assessment of Implementation and Progress

**DOI:** 10.3390/vaccines14060465

**Published:** 2026-05-22

**Authors:** Rosaria Iardino, Danilo Cereda, Simona Scarioni, Elisa Sala, Francesco Cervellera, Sara Russo, Riccardo Vecchio, Maria Virginia Coscarelli, Giuliano Rizzardini, Alessandro Venturi, Luisa Brogonzoli, Catia Rosanna Borriello, Anna Odone

**Affiliations:** 1 Fondazione the Bridge, 20144 Milan, Italy; 2 Directorate General for Health, Lombardy Region, 20124 Milan, Italy; 3 Vaccination Unit, ASST Fatebenefratelli Sacco, 20131 Milan, Italy; 4 Department of Public Health, Experimental and Forensic Medicine, University of Pavia, 27100 Pavia, Italy; 5 Medical Direction, IRCCS Institute Clinici Scientifici Maugeri, 27100 Pavia, Italy; 6 Faculty of Medicine, University Vita-Salute San Raffaele, 20132 Milan, Italy; 7 Centro Studi Bridge for Future, 27100 Pavia, Italy; 8 Fondazione IRCCS Policlinico San Matteo, 27100 Pavia, Italy; 9 Department of Political and Social Sciences, University of Pavia, 27100 Pavia, Italy

**Keywords:** hospital-based vaccinations, life-course immunization, high-risk subjects, regional analysis, healthcare services, Italy

## Abstract

**Background**: In the context of a multi-stakeholder program promoted by Regione Lombardia in collaboration with Fondazione The Bridge and the University of Pavia, the present study investigates the organization and availability of hospital-based vaccination services for high-risk patients. Framing hospitals as strategic hubs for vaccination delivery, the study aimed to map service availability, operational settings and dedicated pathways across the region. **Methods**: A structured questionnaire was administered in 2025 to 40 healthcare organizations, encompassing 114 hospital facilities, including Local Health and Social Care Authorities (ASSTs) and both public and private Scientific Institutes for Research, Hospitalization and Healthcare (IRCCSs). Descriptive and inferential statistical analyses were performed, and findings were compared with those from the 2023 and 2024 editions of the same survey, developed within the “Vaccination—an opportunity for high-risk patients” project, using Pearson’s chi-square test. **Results**: In 2025, 99 facilities (86.8% of respondents) reported providing vaccination services for at-risk individuals. Dedicated vaccination pathways were generally available in more than 50% of facilities for nearly two-thirds of the risk categories considered. Vaccination services for diabetic patients were available in 70.7% of facilities. Among healthcare workers, influenza (93%) and SARS-CoV-2 (89.5%) vaccines were the most frequently offered, with rates approximately ten percentage points higher than those of other vaccines. **Conclusions**: Overall, these findings indicate a regional model progressively consolidating hospital-based vaccination for high-risk groups, with a consistent upward trend in service availability from 2023 to 2025.

## 1. Introduction

High-risk subjects, particularly those with chronic diseases, multimorbidity, and in general immunocompromising conditions, are more susceptible to vaccine-preventable diseases (VPDs) and develop more severe outcomes, including increased hospitalization and mortality [1,2,3]. Vaccination has been shown to be both effective and safe for high-risk subjects [1] and is explicitly prioritized within national immunization policies [4]; however, vaccination uptake in these groups remains frequently suboptimal, partly due to mistrust in vaccines and an increasingly pronounced vaccine hesitancy [5,6].

Building on these persistently suboptimal coverage levels, hospitals have been increasingly recognized as a strategic setting to strengthen vaccination delivery for high-risk subjects [7]. The hospital environment, through the provision of recurrent encounters with high-risk subjects, can enable timely identification of eligible patients and reduce the occurrence of overlooked opportunities for immunization [8]. The COVID-19 pandemic accelerated this transition, highlighting the disproportionate impact of preventable infections among high-risk subjects and the need to incorporate prevention within specialist care pathways [9]. Evidence syntheses indicate that structured hospital-based immunization programs—built on proactive identification of eligible patients, clearly assigned responsibilities, and implementation supports (e.g., standardized procedures, standing orders, and multi-component approaches)—can increase uptake, particularly for priority vaccines such as influenza and pneumococcal vaccination [8,10,11,12].

The degree of support for hospital-based models is found to be strongest in high-complexity clinical areas. In the field of oncology, the heightened risk of VPD-related complications has prompted scientific societies to recommend systematic integration of vaccination into care pathways; moreover, the provision of specialist counselling, accompanied by dedicated pathways, has been associated with improved adherence to recommended vaccinations [13,14]. Recent Italian operational experiences provide further support for the feasibility of in-hospital vaccination counselling and its potential to increase uptake in oncology settings [9,15]. Among patients receiving kidney replacement therapy—especially those on undergoing hemodialysis—the integration of vaccination programs into routine care workflows can improve coverage and has been associated with better clinical outcomes [16]. For transplant candidates and recipients, vaccination management requires specialist hospital-based oversight to account for immune status and ongoing therapies, in line with dedicated guidelines [17]. In the field of rheumatology, the implementation of dedicated pathways and vaccination clinics may facilitate adherence among patients receiving immunomodulatory treatment [18]. The proactive provision of influenza vaccination during hospitalization in high-risk subjects has been associated with favourable signals of increased uptake [19].

In Italy, despite the presence of consolidated recommendations and planning frameworks, implementation of hospital-based vaccination models remains variable [4]. A number of organizational, regulatory, and informational barriers have been identified as potentially hindering the delivery of healthcare in a structured and equitable manner: these include challenges with proactively identifying eligible patients, heterogeneous governance across facilities, constraints related to data management, and the lack of dedicated monitoring systems [4,20]. Digital solutions aimed at enhancing hospital-community integration and information flows are being developed, yet adoption remains uneven [21]. The National Immunization Prevention Plan (“Piano Nazionale di Prevenzione Vaccinale”—PNPV) 2023–2025 reiterates the need for effective and homogeneous strategies and targeted actions for high-risk groups, underscoring the importance of measurable and scalable implementation models across care settings [4].

Despite the existence of supportive evidence and national policy recommendations, systematic and longitudinal evaluations of the organisational implementation of hospital-based vaccination models remain limited.

Within this broader framework, Lombardy provides a particularly pertinent setting for the evaluation hospital-based vaccination pathways for high-risk subjects. As the most populous Italian region, it counts 10,035,500 residents (as of 1 January 2025), including approximately 2.4 million adults aged ≥65 years, i.e., the segment most susceptible to severe outcomes from respiratory vaccine-preventable infections [22]. Lombardy’s hospital network comprises 26 Local Health and Social Care Authorities—ASSTs—and 20 Scientific Institutes for Research, Hospitalization and Healthcare—IRCCSs—(6 public; 14 private), including in total 197 accredited inpatient facilities (105 public; 92 private), reflecting heterogeneous governance and delivery arrangements across the region [23,24]. In this context, respiratory infections targeted by vaccination—such as seasonal influenza, pneumococcal disease, and COVID-19—continue to account for a meaningful burden of severe clinical outcomes and healthcare utilization among older adults and clinically vulnerable groups, reinforcing the need to understand how preventive interventions are operationalised within specialist care settings.

Taken together, these demographic, epidemiological, and structural considerations provide a robust rationale for a systematic mapping of hospital vaccination models, with a focus on organisational variability and integration across care pathways. Since 2023, such an assessment has been conducted within a multi-stakeholder program promoted by Regione Lombardia, in collaboration with the University of Pavia and Fondazione The Bridge, an Operating Research Foundation based in Milan expert in the field of healthcare policies. Findings from this initial assessment highlighted heterogeneity and scope for improvement in standardization and hospital-community integration [25]. Therefore, building on this foundation, the program introduced a yearly survey of hospital vaccination pathways. The aim of the survey was threefold: firstly, to monitor current practices; secondly, to inform organizational improvements; and thirdly, to support regional planning.

The present study reports findings from the 2025 survey and examines trends compared with previous editions.

## 2. Materials and Methods

### 2.1. Study Setting

In the context of the project “VaccinAzione: an opportunity for health for frail populations in Lombardy” (*VaccinAzione* means Vaccination & Action), promoted since 2023 by Fondazione The Bridge in collaboration with the Prevention Unit of the Directorate General for Welfare of the Lombardy Region, multistakeholder activities were planned and implemented to increase vaccination coverage among high-risk population groups. The objective of the interventions was to identify issues relating to the area of prevention, emphasise unmet health needs, highlight best practices, and foster the adoption of targeted improvement actions.

In 2025, in continuity with the program, the following activities were carried out: discussions among scientific societies on the integration of vaccination within care pathways for frail individuals; continuation of the regional communication campaign *#DIRITTOALVACCINO* (in which “*diritto*” means “right” and “*al vaccino*” means “to vaccination”), targeting both frail individuals and healthcare professionals; and a regional event promoted by the Lombardy Region, during which strategic issues were discussed with healthcare institutions, healthcare companies and patient associations, and Lombardy healthcare facilities recording the highest number of vaccinations administrated in the previous year were awarded. Within this framework, the third edition of the regional survey was implemented with the objective of mapping hospital-based vaccination models and pathways for frail individuals in the Lombardy context. The full text of the survey is available as Supplementary Material File S1.

### 2.2. Study Design and Data Collection

Since 2023, a working group composed of Fondazione The Bridge, the University of Pavia and the Directorate-General for Welfare of the Lombardy Region, has conducted a cross-sectional observational study using a structured questionnaire. The questionnaire was originally developed and administered in 2023 and subsequently revised in 2024 and again in 2025. The survey tool was informed by the appraisal of available scientific evidence on hospital-based vaccination models, by outputs from project seminars promoted by the Focus Group, and by consultation with experts in the field.

The survey methods used in the year 2025 present significant differences with those employed in the preceding two years of administration, particularly with regard to the samples included in the analyses, despite all focusing on facilities within the Lombardy Regional Health Service involved in the care of frail patients and in the implementation of hospital-based activities aimed at promoting vaccine uptake.

In fact, in 2023 and 2024, the surveys included a wider sample, investigating all healthcare facilities in Lombardy, including ASSTs (*Aziende Socio-Sanitarie Territoriali*: Territorial Health and Social Care Authorities are public organizations in Lombardy that run hospitals, clinics, and local healthcare services. They provide everyday healthcare for citizens, such as medical visits, hospital care, and emergency services), public and private IRCCSs (*Istituti di Ricovero e Cura a Carattere Scientifico*: Scientific Institutes for Research, Hospitalization and Healthcare are highly specialized hospitals that treat patients and also conduct advanced medical research, usually focused on specific diseases or medical fields), and accredited private facilities. However, the analyses focused on institutional-level responses rather than facility-level responses.

In 2025, the survey changed its scope to include only public and private ASSTs and IRCCSs, excluding accredited private institutions. The methodological approach changed substantially because a single response per institution was no longer sufficient; all the individual facilities belonging to each entity were surveyed. Overall, the unit of analysis was mainly the institution as a whole in 2023 and 2024, with data aggregated at the organizational level, while in the last year, the unit of measurement is represented by the individual facility. This resulted in a substantial increase in the number of units that provided a response, as well as a more comprehensive array of information.

In order to ensure comparability of results, the following analysis is based solely on ASSTs and IRCCSs, with accredited private facilities excluded from the 2023 and 2024 samples. This methodological decision is underpinned by the principle of consistency with previous surveys, thereby allowing to compare the three years surveyed.

In the 2025 iteration of the survey, there were also differences in the mode of survey administration. In earlier editions, the survey was disseminated by the Prevention Unit of the Directorate General for Welfare of the Lombardy Region to all hospital medical directorates, requesting the completion of the survey online on a dedicated digital platform (IdSurvey, IdWeb s.r.l., Città di Castello, Italy; available online: https://www.idsurvey.com), a web-based program specifically designed for the management of research, surveys, and customer satisfaction studies that uses the Computer-Assisted Web Interviewing (CAWI) technique, and therefore completed independently. To improve the accuracy of the responses, in 2025, the Prevention Unit of the Directorate General for Welfare arranged scheduled telephone appointments with all hospital medical directorates in Lombardy, during which the survey was completed by the respondents with the assistance of two medical residents from the Hygiene Unit of the Department of Public Health, Experimental and Forensic Medicine at the University of Pavia. The interviewers were specifically trained to conduct standardised interviews to increase the consistency of question interpretation and to ensure greater homogeneity in data collection.

Overall, for 2025, 40 healthcare organizations were surveyed, corresponding to 114 hospital facilities. Local Health and Social Care Authorities (ASSTs) and both public and private Scientific Institutes for Research, Hospitalization and Healthcare (IRCCSs) were surveyed.

### 2.3. Structure of the Survey

The questionnaire opened with a preliminary section deigned to characterize the participating healthcare facility (ASST; public IRCCS; private IRCCS) and the respondent’s professional role (Medical Director, Director of the Medical–Surgical Department, physician affiliated with the Medical Directorate or the Department, other).

A dichotomous screening question was used to investigate the presence of structured vaccine-promotion activities targeting frail individuals. In the event of a positive response, the organizational characteristics of hospital-based vaccination models were explored, with specific reference to counselling and administration settings and the healthcare professionals involved (in inpatient wards, specialist outpatient clinics, or community settings). Vaccination recording practices are also assessed, with particular attention to the use of the regional computerised vaccination registry (ARVAX) and the training of dedicated staff.

The subsequent section addressed the availability of fee-based vaccinations, the existence of standard operating procedures, and the extent to which vaccination pathways were integrated into Clinical Pathways (PDTAs: Diagnostic and Therapeutic Care Pathways) for selected categories of frail patients. Finally, the questionnaire included items aimed at identifying the main risk groups for which hospital-based vaccination pathways were active and at describing vaccination provision for healthcare workers, including information on vaccine availability and staff uptake of seasonal influenza vaccination.

### 2.4. Data Analysis

Data were summarized using descriptive statistics to characterize frequency patterns and distributions. To explore whether organizational differences between hospital settings could influence the findings, facilities were stratified by type (ASST, public IRCCS, and private IRCCS) and analyses were conducted within each stratum. No relevant effect of facility type on vaccination practices emerged, as differences across groups were small and not statistically significant.

All analyses were conducted using IBM SPSS Statistics (Version 28, IBM Corp., Armonk, NY, USA).

For the purposes of this article, only the most informative results are presented, with particular emphasis on comparisons with data from previous years whenever available. Differences across survey years were assessed using Pearson chi-square tests of independence (df = 2). For items allowing multiple responses, tests were conducted separately for each response option. Post hoc pairwise comparisons were performed with Bonferroni correction (k = 3 pairs). Statistical significance was set at *p* < 0.05.

## 3. Results

### 3.1. Distribution of Facilities

In Lombardy, the hospital network—not counting private accredited institutions—includes 27 ASSTs and 20 IRCCSs (with IRCCS San Gerardo of Monza performing both functions); together, the 46 entities oversee a total of 127 hospital facilities.

In 2025, 40 of the 46 identified institutions participated in the survey, covering 87% of eligible institutions, with a total of 114 out of 127 facilities (89% in terms of eligible facilities). Most responses came from ASST facilities (86%; N = 98), followed by private IRCCSs (9%; N = 10) and public IRCCSs (5%; N = 6). As the survey achieved an 89% facility-level response rate and covered 87% of eligible institutions, the sample can be considered highly representative of the total population of hospital facilities in Lombardy.

### 3.2. Contextual Denominators

As previously mentioned, in the two years, the survey targeted ASSTs and IRCCSs only at the institutional level, with one response collected per organization. In 2023, participation included 23 of 27 ASSTs and 17 of 20 IRCCSs, yielding 40 total responses (87% of the total). In 2024, the same institutions were surveyed, and responses were primarily aggregated at the organizational level. ASSTs generally provided a single consolidated response, while multiple responses from IRCCSs were merged. The final sample comprised 26 ASSTs and 17 IRCCSs, for a total of 43 responses (93% of the total).

Although the coverage in 2023 and 2024 also exceeded 87% of regional institutions, it is important to note that the different methodology adopted in 2025, with data collected at the facility rather than institutional level, enabled a more detailed, granular, and comprehensive analysis. Consequently, the 2025 findings provide a more robust and precise representation of vaccination practices across individual facilities. Earlier surveys remain informative, but they reflect institutional-level trends rather than facility-specific characteristics.

For these reasons, overall, the survey included 114 respondents in 2025, 43 in 2024, and 40 in 2023.

### 3.3. Offer of Vaccination for High-Risk Patients

In the 2025 survey, a total of 99 facilities stated that they offered vaccination services for at-risk individuals, accounting for 86.8% of the total number of responses. Conversely, 15 facilities reported not offering this service.

A subsequent analysis of the distribution by type of institution reveals that the availability of vaccinations is particularly high among ASSTs (approximately 88%), followed by public IRCCSs (83%) and private IRCCSs (80%), indicating a fairly even distribution of the service across the various institutional settings.

A comparison with previous surveys reveals a substantially stable picture compared to 2024, when the percentage of facilities that offer vaccination to at-risk patients was 86%, and suggests an increase compared to 2023 (77.5%).

Despite the evident increase in the proportion of facilities offering vaccination across the three-year period, this trend did not reach statistical significance (χ^2^(2) = 2.07, *p* = 0.355). This finding suggests that the observed improvement reflects a consistent and stable pattern rather than a statistically abrupt change.

The comparison between the three years is presented in Table 1.

The following results—with the exception of where otherwise specified—refer to the 99 hospitals that reported in the 2025 survey to offer vaccinations to vulnerable patients, irrespective of the specific type. Similarly, for the years 2023 and 2024, the responses reported correspond to 31 and 37 entities that provided a vaccination offer to at-risk patients.

### 3.4. Offer Within the Hospital

In order to ascertain the context in which vaccination was offered within the healthcare facility, respondents were asked to provide information regarding the specific circumstances in which it was administered within the hospital setting. The provision of the vaccine occurs during hospitalization in 82.8% of the facilities, and in 72.7% during the specialist visit.

**Figure 1:**
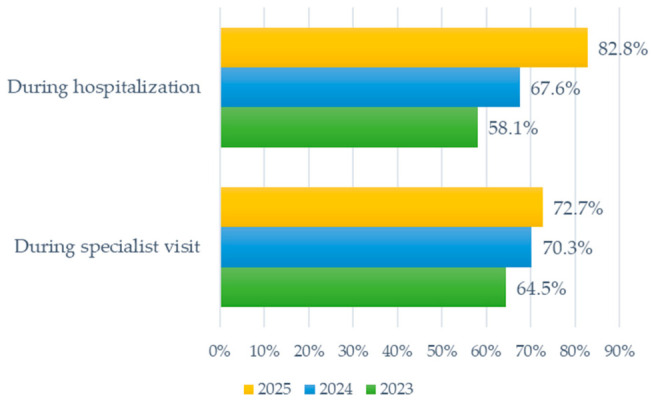
Context of vaccination offers within the hospital; percentage of the respondents. Note. Denominators: 2023 N = 31; 2024 N = 37; 2025 N = 99.

A comparison of the data from previous years (see Figure 1) reveals a consistent upward trend in the provision of both services over the three-year period from 2023 to 2025.

Specifically, the proportion of vaccination performed during the specialists’ visits increased from 64.5% in 2023 to 70.3% in 2024, and it reached 72.7% in 2025, indicating a steady yet moderate increase, which did not reach statistical significance (χ^2^(2) = 0.77, *p* = 0.680).

A comparable yet more pronounced trend is observed in the context of vaccination during hospitalization, exhibiting an increase from 58.1% in 2023 to 67.6% in 2024 and 82.8% in 2025; this increase was statistically significant (χ^2^(2) = 9.03, *p* = 0.011), with post hoc pairwise comparisons indicating that the difference was primarily driven by the increase between 2023 and 2025 (*p* = 0.013), while 2023 and 2024 did not differ significantly.

Overall, vaccination during hospitalization show the most significant increase in absolute terms, with a rise of more than 24 percentage points across the three-year period, suggesting a significant increase in hospital capacity.

### 3.5. Offer by Category of Risk

The availability of dedicated vaccination pathways for at-risk groups was generally favorable. In 2025, approximately two-thirds of the categories were covered by more than 50% of facilities offering a specific vaccination pathway, indicating widespread provision and attention to the needs of several at-risk populations.

The most prevalent categories in 2025 were as follows: chronic heart disease (65.7%), chronic lung disease and pregnancy (63.6%), diabetes mellitus and chronic renal/adrenal insufficiency (62.6%), onco-hematological diseases and malignant neoplasms (58.6%), asplenia or candidates for splenectomy (56.6%), neurological diseases (55.6%), congenital or acquired immunodeficiencies (54.5%), HIV infection and individuals over 65 years of age (53.5%), autoimmune diseases (52.5%), and chronic liver disease (51.5%).

Chi-square tests of independence were conducted separately for each at-risk category to assess whether coverage changed significantly across the three survey years (multiple responses allowed). Of the 22 categories present across all three years, seven showed a statistically significant increase: haemoglobinopathies (*p* < 0.001), patients with cerebrospinal fluid leaks (*p* = 0.003), chronic inflammatory diseases (*p* = 0.004), cochlear implant recipients (*p* = 0.013), chronic heart disease (*p* = 0.022), chronic alcoholism (*p* = 0.034), and autoimmune diseases (*p* = 0.044).

Table 2 offers a comparative analysis of the percentage of entities that offer a vaccination pathway by category of risk.

Certain categories are very prominent across all three years: these categories include, but are not limited to, chronic renal/adrenal insufficiency, chronic lung diseases, diabetes mellitus, pregnancy, and onco-haematological diseases. This observation supports the prevailing notion that these categories are associated with a heightened risk, and consequently, many hospitals have a vaccination path dedicated to them.

A gradual increase in percentages over time is observed, particularly evident in 2025, when almost all categories exceeded 50% of recorded respondents that have the provision of a vaccination path for them. In 2025, there is also a strengthening of the recognition of additional vulnerable groups, such as chronic heart disease, congenital or acquired immunodeficiencies, HIV infection, immunosuppression, and chronic inflammatory diseases, which show significant increases both in percentage and ranking position.

Conversely, lower percentages are observed for highly specialized or infrequent categories, such as cochlear implants, cerebrospinal fluid leakage, and chronic alcoholism. These categories are likely associated with reduced prevalence or awareness.

Overall, the ranking reveals a discernible shift towards a more expansive and inclusive understanding of risk. This shift is accompanied by an enhancement in sensitivity towards protecting the most vulnerable populations with vaccines.

### 3.6. Offer for Patients with Diabetes

The survey subsequently focused on the offer for individuals with diabetes, a high-risk population for whom immunization is strongly recommended due to the increased risk of severe complications from vaccine-preventable diseases. In addition, diabetes is widely prevalent across age groups and genders.

Assessing the presence of a vaccination offer for the specific category of diabetic patients, in 2025 it is present in 70.7% of the facilities.

**Figure 2:**
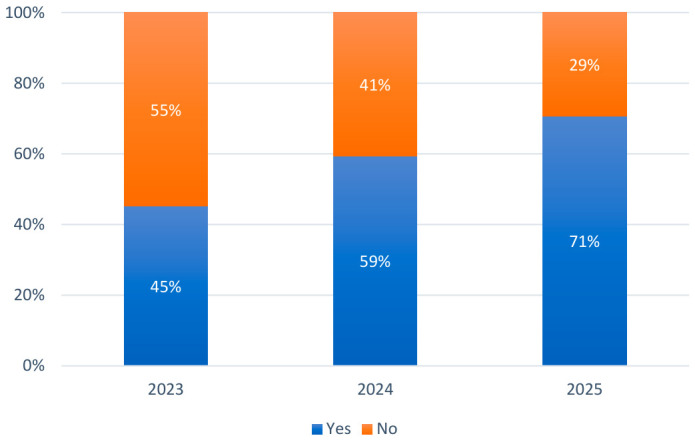
Vaccination offers for diabetic patients over the years in percentages. Note. Denominators: 2023 N = 31; 2024 N = 37; 2025 N = 99.

The percentages are lower for the other years under consideration, particularly for 2023, when institutions without a dedicated vaccination offer for diabetic patients outnumber those with one (54.8% vs. 45.2%, respectively) (see Figure 2).

Between 2023 and 2024, the number of institutions with a dedicated vaccination program for diabetic patients grew by almost 15 percentage points, and in 2025 by another ten percentage points, indicating a total increase of more than 25 percentage points during the observed period. A statistically significant association between year and the presence of vaccination programs for diabetic patients was observed (χ^2^(2) = 6.975, *p* = 0.031).

### 3.7. Offer for Healthcare Workers

For 2025, the survey also assessed the willingness of facilities to promote vaccination to healthcare workers (HCWs).

NOTE: This question was addressed to all facilities, including those that did not provide vaccinations to vulnerable patients; therefore, the findings refer to the entire sample of 114 facilities.

**Figure 3:**
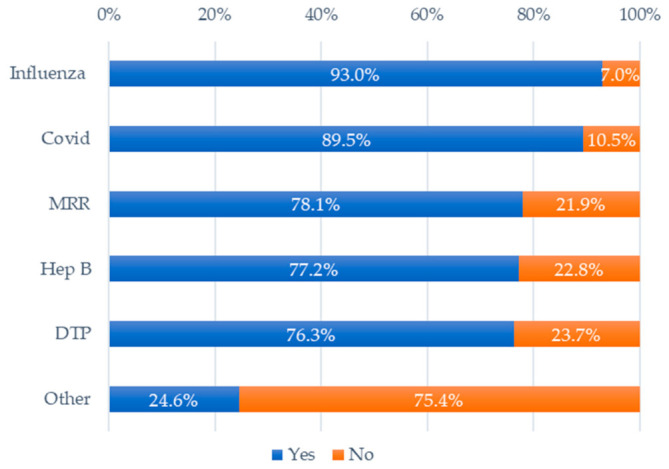
Vaccination offers dedicated to healthcare personnel by vaccine, 2025, in percentages.

Influenza (93%) and SARS-CoV-2 (89.5%) vaccines were the most frequently available for HCWs, with offer rates approximately ten percentage points higher than those observed for other vaccines. Intermediate levels of availability were reported for MMR (78.1%), hepatitis B (77.2%), and DTP (76.3%). Although these values remain relatively high, they lag behind influenza and SARS-CoV-2 coverage, and one-fifth of facilities indeed do not offer these routine vaccinations. Other vaccines (e.g., HPV or herpes zoster) were available in only 24.6% of facilities. The percentages are visible in Figure 3. This pattern likely reflects well-established practices, further strengthened by targeted information campaigns and a heightened perception of occupational risk.

Overall, the results indicate that vaccines traditionally prioritized in prevention strategies are widely accessible, whereas substantial gaps persist for other immunizations.

## 4. Discussion

The present survey provides a regional organizational mapping of the reported availability of hospital-based vaccination services for high-risk subjects in Lombardy. In 2025, the majority of participating facilities reported offering vaccination services for at-risk individuals, suggesting that hospitals are increasingly involved in preventive activities for clinically vulnerable groups. This finding may be of interest, particularly when compared with the broader literature, which identifies underutilization of opportunities to assess vaccination status, provide counselling, and reduce missed vaccinations among high-risk adults through hospitalization, discharge, and specialist encounters [7,8,10,11,12,19]. The potential role of hospital-based pathways may also extend beyond the individual patient. In accordance with recommendations for selected high-risk groups, the provision of counselling in specialist settings can support cocooning strategies involving caregivers and household contacts, particularly when these individuals contribute to the protection of immunocompromised or at-risk individuals [26]. Future organizational models should therefore consider whether and how caregiver counselling can be incorporated into hospital-based prevention pathways.

Within this framework, structured vaccination pathways represent a significant component of health-system preparedness. The global experience of the COVID-19 pandemic showed that immunization programs supported by established delivery infrastructures, trained personnel, clear governance, and reliable information systems can respond more rapidly during public health emergencies [27]. Strengthening hospital-based vaccination models should therefore be viewed not only as a disease-specific intervention, but also as part of a broader strategy to protect vulnerable populations and improve health-systems resilience.

Survey items on operational settings indicate a growing share of counselling and administration in inpatient wards and specialist outpatient clinics, while a significant proportion remains through territorial pathways. The aforementioned model is predicated on a multifaceted, integrated delivery paradigm, wherein hospital departments and specialist clinics proactively identify and expeditiously offer services during clinical interactions, whereas territorial services and primary care channels ensure continuity and scale. This complementary role persists even as inpatient vaccinations rose from 58.1% to 82.8% between 2023 and 2025 and outpatient specialist visits reached 72.7%. The implementation evidence suggests that multi-setting models improve uptake only when responsibilities, referral and feedback loops, and monitoring mechanisms are explicitly designed and governed, rather than left to local ad-hoc practice [8,20,21].

Importantly, the reported near-universal use of the regional immunization information system suggests an important organizational prerequisite for coordination and monitoring. A shared registry supports timely documentation, access to vaccination history and operational accountability across hospital-community interfaces. Immunization information systems are widely recognized as being fundamental to monitor coverage, enable reminder/recall interventions and support audit/feedback cycles-particularly in adult and high-risk vaccination programs where provider fragmentation is common [28,29].

The reported availability of category-specific pathways was found to be more prevalent for common chronic conditions, including cardiovascular disease, chronic respiratory disease, renal disease, and diabetes. This phenomenon was also observed for several immunocompromising conditions. This finding aligns with the existent literature, which demonstrates that patients with chronic diseases, cancer, transplant-related conditions, and immune-mediated disorders are priority groups for vaccination and may benefit from vaccination assessment embedded into specialist care pathways [13,14,15,16,17,18]. Diabetes is particularly informative because of its high prevalence and frequent contact with both outpatient specialist services and inpatient care. However, the presence of a dedicated offer should not be interpreted as evidence of a systematic delivery of vaccination, nor of an increase in uptake.

The widespread reported provision of influenza vaccination to HCWs suggests that hospitals may represent an important setting for the enhancement of occupational immunization strategies. Improving uptake among HCWs likely requires multi-component interventions, including on-site access, active promotion, reminder systems, leadership endorsement, audit and feedback mechanisms, and supportive organizational norms, which have shown greater effectiveness than single interventions [23,24]. In addition to safeguarding against occupational hazards, strengthening HCW vaccination has the potential to bolster the credibility and uniformity of counselling imparted to vulnerable patients within clinical pathways, particularly in contexts where mistrust and hesitancy remain barriers [5,6]. Despite the expansion of the offer, the institutionalization of vaccination within clinical pathways remains incomplete, indicating that prevention is not yet systematically treated as an integral component of the care plan across facilities. The transition from PDTA to “PPDTA” (Preventive, Diagnostic and Therapeutic Care Pathways) requires governance and implementation mechanisms that operationalize responsibilities, embed standardized triggers at points of care (ward admission/discharge; specialist visits) and adopt measurable indicators integrated into routine monitoring. Integrated-care frameworks emphasize that prevention becomes effectively embedded only when supported by cross-setting coordination, shared information flows and accountability mechanisms [29].

### Study Limitations

This study is subject to several limitations.

First, the sample and unit of analysis differed across survey editions: in 2023 and 2024, data were mainly collected at the institutional level, whereas in 2025, the survey was administered at the facility level and focused on ASSTs and public and private IRCCSs, excluding non-IRCCS accredited private facilities. This limits direct longitudinal comparability.

Second, the administration mode changed in 2025 from self-administered online questionnaires to assisted telephone interviews, which may have introduced interview-mode effects or social desirability bias.

Third, data were self-reported by hospital medical directorates and were not independently validated through audits or patient-level vaccination registries. Therefore, the present survey provides a detailed assessment of the presence and organizational characteristics of hospital-based vaccination pathways, but does not measure actual vaccination uptake, completion of multidose schedules, timeliness, equity of access, or health outcomes.

Finally, the statistical analysis was mainly descriptive, limiting the possibility of identifying determinants of implementation or assessing the effectiveness of specific organizational models.

These limitations should be considered when interpreting the findings as a regional organizational mapping rather than as an evaluation of vaccination coverage or program impact.

## 5. Conclusions

The findings may support improvement initiatives by offering a framework for defining minimum organizational standards, promoting the integration of vaccination assessment and counselling into routine clinical pathways, and encouraging the use of shared indicators to monitor implementation across facilities and risk groups. From this standpoint, the engagement of regional institutional networks (e.g., oncology networks) could be considered a pivotal mechanism to facilitate the systematic integration of prevention-oriented pathways (PPDTAs) within prevailing organizational frameworks. Strengthening collaboration with scientific societies could further enhance the alignment with clinical guidelines and support the integration of vaccination into specialist care pathways Furthermore, the active involvement of patient associations has the potential to enhance awareness, address vaccine hesitancy, and promote uptake among high-risk groups and their caregivers. Future policy efforts should link organizational survey data with immunization registry and patient-level indicators, in order to enable the evaluation of vaccination uptake, completion of schedules, equity of access, and the real impact of hospital-based vaccination models.

## Figures and Tables

**Table 1:** Presence of vaccination offers for high-risk patients; number of respondents and percentages per year (institution-level in 2023–2024 vs. facility-level in 2025, not directly comparable).

	2023		2024		2025	
	n	%	n	%	n	%
No	9	22.5%	6	14.0%	15	13.2%
Yes	31	77.5%	37	86.0%	99	86.8%
Total	40	100%	43	100%	114	100%
χ^2^(2)	2.07					
*p*-value	0.355 (n.s.)					

Note. Denominators: 2023 N = 40; 2024 N = 43; 2025 N = 114. χ^2^(2) = 2.07, *p* = 0.355. The results from the 2023 and 2024 surveys are provided at the institution level, while for 2025, they are provided at the facility level; therefore, these are not directly comparable.

**Table 2:** Vaccination pathway offers over the years by category of risk (alphabetical order).

Risk Category	2023	2024	2025	*p*
Anatomical or functional asplenia/splenectomy candidates	41.90%	37.80%	56.60%	0.096
At-risk infants/children	n.d.	n.d.	50.50%	-
Autoimmune diseases	38.70%	29.70%	52.50%	0.044
Chronic alcoholism	16.10%	16.20%	34.30%	0.034
Chronic heart disease	45.20%	43.20%	65.70%	0.022
Chronic inflammatory diseases	29.00%	21.60%	50.50%	0.004
Chronic liver diseases	41.90%	32.40%	51.50%	0.126
Chronic lung disease	58.10%	54.10%	63.60%	0.569
Chronic renal/adrenal insufficiency	64.50%	59.50%	62.60%	0.906
Conditions requiring long-term immunosuppressive treatment	29.00%	40.50%	50.50%	0.096
Congenital or acquired immunodeficiencies	41.90%	37.80%	54.50%	0.160
Diabetes mellitus	54.80%	51.40%	62.60%	0.440
Haemoglobinopathies	12.90%	16.20%	43.40%	<0.001
HIV infection	35.50%	40.50%	53.50%	0.138
Individuals over 65 years of age	48.40%	40.50%	53.50%	0.397
Individuals with cerebrospinal fluid leakage due to trauma or surgery	16.10%	8.10%	34.30%	0.003
Malignant neoplasms	51.60%	48.60%	58.60%	0.532
Neurological diseases	45.20%	37.80%	55.60%	0.158
Onco-haematological diseases	51.60%	51.40%	58.60%	0.661
Organ and bone marrow transplantation	32.30%	40.50%	48.50%	0.257
Other	n.d.	18.90%	5.10%	-
Patients referred to STI services	25.80%	27.00%	43.40%	0.082
Pregnancy	51.60%	56.80%	63.60%	0.446
Presence of cochlear implant	12.90%	13.50%	33.30%	0.013
Rare diseases	n.d.	n.d.	36.40%	-

Note: Multiple responses allowed. Denominators: 2023 N = 31; 2024 N = 37; 2025 N = 99. In all significant cases, post hoc pairwise comparisons with Bonferroni correction indicated that differences were primarily driven by an increase between 2024 and 2025, while 2023 and 2024 did not differ significantly. Given the large number of simultaneous tests performed (k = 22), results should be interpreted with appropriate caution; applying a Bonferroni-corrected threshold (*p* < 0.0023) would retain only haemoglobinopathies, patients with cerebrospinal fluid leaks, and chronic inflammatory diseases as statistically significant.

## Data Availability

The data that support the findings of this study are not publicly available due to privacy restrictions. Data may be available from the corresponding author upon reasonable request and subject to institutional approval.

## Supplementary Materials

The following supporting information can be downloaded at: https://www.mdpi.com/article/10.3390/vaccines14060465/s1, File S1: Regional survey for the mapping of the vaccination offer for high-risk patients in Lombardy (english translation).

## Abbreviations

The following abbreviations are used in this manuscript:

ASST	Local Health and Social Care Authority
IRCCS	Scientific Institute for Research, Hospitalization and Healthcare
CAWI	Computer-Assisted Web Interviewing
PDTA	Diagnostic and Therapeutic Care Pathway
DTP	Diphtheria Tetanus Pertussis vaccine
Hep B	Hepatitis B vaccine
MRR	Measles Mumps Rubella vaccine
