# Supplementary material for: Hospital-Based Models of Immunization for High-Risk Subjects in Lombardy (Italy): A Region-Wide Assessment of Implementation and Progress
Source: Vaccines (Basel). 2026 May 22;14(6):465. doi: 10.3390/vaccines14060465 (PMC13307903; doi:10.3390/vaccines14060465)
Supplement: Supplementary file 1 [file vaccines-14-00465-s001.zip › vaccines-4288673-File S1.pdf]

Supplementary material. Regional Survey for the Mapping of the Vaccination Offer for High-Risk Patients in Lombardy (english translation)

HOSPITAL VACCINATION MODELS AND PATHWAYS FOR HIGH-RISK INDIVIDUALS IN LOMBARDY: A REGIONAL SURVEY

The Bridge Foundation, with the support of the University of Pavia and in collaboration with the Lombardy Region, is running a project aimed at promoting vaccination as a public health measure for vulnerable individuals.

For this reason, we ask you to contribute by completing a very short questionnaire (estimated time to complete: 5–10 minutes).

The questions aim to investigate **the availability and characteristics of hospital vaccination services**, integrated into the care pathways for various categories of vulnerable patients.

The aim of this survey is to map out the vaccination provision models for vulnerable individuals in the inpatient and care facilities of ASSTs (TN: Local Health and Social Care Trusts) and IRCCSs (TN: Scientific Institutes for Research, Hospitalization and Healthcare) in the Lombardy Region.

The data you provide will not be disclosed to third parties, and the survey results will only be published in aggregated form.

We thank you in advance for your valuable contribution.

0.1) Type of entity:

1. Local Health and Social Care Trust (ASST)
2. **Public** Scientific Institute for Research, Hospitalization and Healthcare (IRCCS)
3. **Private** Scientific Institute for Research, Hospitalization and Healthcare (IRCCS)

0.2) Name of the ASST/IRCCS:

0.2.1 Name of the facility on behalf of which you are responding. ***Please note: you will need to complete a questionnaire for each facility on behalf of which you are responding:***

0.3) Role of the interviewee

1. Medical Director
2. Director of the Hospital Medical Directorate
3. Clinician belonging to the Medical Directorate or to Hospital Medical Directorate
4. Other: ____________

1. **Are vaccines being offered to high-risk individuals at the facility?**
    1. Yes. Please continue to complete the questionnaire
    2. No.

2. **How is vaccination counselling provided for vulnerable individuals?**
   Multiple choice

    1. Within the hospital ward
    2. Within the facility
    3. In the community (vaccination centres, community care centres, counselling centres, etc.)
    4. Other:_______

3. **How are vaccines administered to vulnerable individuals?**
   Multiple choice
    1. Within the ward during hospitalisation
    2. Within the facility (in a specialty or vaccination clinic)
    3. In the community (vaccination centres, community care centres, counselling centres, etc.)
    4. Other:_______

   3.1 Who is actively involved in administering the vaccine?
   Multiple choice

    1. Specialist doctor/healthcare professional belonging to the hospital ward
    2. Specialist doctor/healthcare professional belonging to a specialist department, working in an inpatient setting (e.g., diabetologist, nephrologist…)
    3. Specialist doctor/healthcare professional in preventive care (e.g., public health doctor, healthcare assistant or nurse…)

4. **Which software is used to record vaccinations for vulnerable individuals?**
   Multiple choice
    1. Regional Informatics Register SIAVR/ARVAX
    2. Data on vaccine administration is sent to the territory for registration
    3. Other methods of registration. Please specify: ___
    4. Not recorded, but only noted in the medical record

5. **The Lombardy Region has launched a new vaccination software system, ARVAX, which enables the recording of all vaccinations. Has the healthcare staff responsible for recording vaccinations been trained to use the new vaccination software?**
    1. Yes, they have been trained and are currently using it
    2. Yes, they have been trained but are not yet using it
    3. No, but they are due to start during the autumn vaccination campaign
    4. No

6. **Is vaccination available during hospitalization?**
    1. Yes
    2. No

7. **Is vaccination available during specialistic visits?**
    1. Yes
    2. No

8. **Are paid vaccinations available at the hospital?**
    1. Yes
    2. No

    8.1 If so, which vaccines and for which population targets?

Vaccine	Target population

9. **For which of the following categories of health risk has a hospital vaccination programme been established?**

Multiple choice

1. Chronic heart disease
2. Chronic lung disease
3. Diabetes mellitus
4. Neurological disorders
5. Pregnancy
6. Chronic liver disease (including cirrhosis and progressive chronic alcohol-related liver disease)
7. Chronic alcoholism
8. Patients with cerebrospinal fluid leaks due to trauma or surgery
9. Presence of a cochlear implant
10. Haemoglobinopathies such as sickle cell anaemia and thalassaemia
11. Congenital or acquired immunodeficiencies
12. HIV infection
13. Patients referred to STI services
14. Anatomical or functional asplenia and patients scheduled for splenectomy
15. Onco-haematological disorders (leukaemias, lymphomas and multiple myeloma)
16. Malignant neoplasms
17. Organ and bone marrow transplants
18. Conditions requiring long-term immunosuppressive treatment
19. Chronic renal/adrenal failure (including dialysis patients)
20. Autoimmune diseases
21. Chronic inflammatory diseases
22. Rare diseases
23. People over 65 years of age
24. At-risk infants/children
25. Other. Please specify: ___

10. **Is there a specific procedure in place within the facility for managing the vaccination pathway for vulnerable patients (vaccination history, counselling and vaccination offer)?**
    1. Yes
    2. No

    10.1 If so, when was it last updated? (year)

    ____

11. **For which categories of high-risk patients under the care of the facility has a disease-specific vaccination programme been included in the PDTA (Diagnostic, Therapeutic and Care Pathway)?**

Multiple choice

1. Chronic heart disease
2. Chronic lung disease
3. Diabetes mellitus
4. Chronic liver disease (including cirrhosis and progressive chronic alcohol-related liver disease)
5. Chronic alcoholism
6. Patients with cerebrospinal fluid leaks due to trauma or surgery
7. Presence of a cochlear implant
8. Haemoglobinopathies such as sickle cell anaemia and thalassaemia
9. Congenital or acquired immunodeficiencies
10. HIV infection
11. Anatomical or functional asplenia and patients scheduled for splenectomy
12. Onco-haematological disorders (leukaemias, lymphomas and multiple myeloma)
13. Malignant neoplasms
14. Organ and bone marrow transplantation
15. Conditions requiring long-term immunosuppressive treatment
16. Chronic renal/adrenal insufficiency
17. Other. Please specify: ___
18. None

12. **Has a vaccination programme been organised for diabetic patients?**

    1. Yes
    2. No

13. **Has a vaccination programme been organised for patients on dialysis?**

    1. Yes
    2. No

14. **Has a vaccination programme been organised for pregnant women?**

    1. Yes
    2. No

15. **If IRCCS, how are vaccines supplied to your facility? This refers to vaccines intended for vulnerable individuals for whom a free regional offer is available.**

1. Independent: the hospital is entirely responsible for procurement (e.g. through the hospital pharmacy service)
2. Interdependent: procurement is carried out by another organisation (e.g. ASST)

16. **Does the hospital offer a vaccination programme specifically for healthcare staff?**

**Vaccine**		*Multiple choice*
**16.1 Flu**	1. Yes	1. Administered by the occupational health service
		2. Administered by the hospital vaccination clinic

		3. Administered on-site (in wards, specialist clinics, etc.)
	2. No	
**16.2 Covid**	1. Yes	1. Administered by the occupational health service
		2. Administered by the hospital vaccination clinic
		3. Administered on-site (in wards, specialist clinics, etc.)
	2. No	
**16.3 MMR**	1. Yes	1. Administered by the occupational health service
		2. Administered by the hospital vaccination clinic
		3. Administered on-site (in wards, specialist clinics, etc.)
	2. No	
**16.4 Hepatitis B**	1. Yes	1. Administered by the occupational health service
		2. Administered by the hospital vaccination clinic
		3. Administered on-site (in wards, specialist clinics, etc.)
	2. No	
**16.5 DTaP**	1. Yes	1. Administered by the occupational health service
		2. Administered by the hospital vaccination clinic
		3. Administered on-site (in wards, specialist clinics, etc.)
	2. No	
**16.6 Other (please specify): ______**	1. Yes	1. Administered by the occupational health service
		2. Administered by the hospital vaccination clinic
		3. Administered on-site (in wards, specialist clinics, etc.)
	2. No	

16.1.1 If so, what has been the approximate adoption rate among healthcare workers for the flu vaccine over the past year?

1. 0 - 10%
2. 11 - 30%
3. 31 - 70%
4. >71%
